# Prior incarceration, restrictive housing, and posttraumatic stress disorder symptoms in a community sample of persons who use drugs

**DOI:** 10.1186/s40352-024-00276-7

**Published:** 2024-04-26

**Authors:** James A. Hammock, Teresa López-Castro, Aaron D. Fox

**Affiliations:** 1 Albert Einstein College of Medicine, 1300 Morris Park Ave, Bronx, NY 10461 USA; 2 The City College of New York, City University of New York, 160 Convent Ave, New York, NY 10031 USA; 3 Montefiore Medical Center, 111 E 210th St, Bronx, NY 10467 USA

**Keywords:** PTSD, Solitary confinement, Restrictive housing, People who use drugs, Substance use treatment, Opioid use disorder

## Abstract

**Background:**

Criminalization of drugs in the United States (US) has extensive consequences for people who use drugs (PWUD). Incarceration and substance use overlap with 65% of the US prison population meeting substance use disorder (SUD) criteria. Exposure to the criminal-legal system negatively impacts the health of PWUD. PTSD is commonly comorbid with SUDs, and exposure to restrictive housing (RH) during incarceration may worsen mental health. Because PWUD are disproportionately incarcerated, experiences occurring during incarceration, such as RH, may contribute to the development or exacerbation of PTSD and SUDs. This study of PWUD investigated prior criminal-legal system exposure and its association with PTSD symptoms in community-dwelling PWUD.

**Methods:**

This cross-sectional study recruited PWUD from syringe service programs (SSP). Inclusion criteria were: age 18+, current or past opioid use disorder, and SSP enrollment. Data collected included: sociodemographics; incarceration, substance use, SUD treatment history, and PTSD assessments (Life Events Checklist for DSM-5 and the PTSD Checklist for DSM-5). Bivariate testing and multivariate logistic regression analyses, with probable PTSD as the dependent variable and a three-level variable for criminal legal history as the independent variable, were conducted to determine whether incarceration and RH were associated with probable PTSD.

**Results:**

Of 139 participants, 78% had an incarceration history with 57% of these having a history of RH. 57% of participants screened positive for probable PTSD, and physical assault was the most common traumatic exposure. Any history of incarceration was not associated with probable PTSD diagnosis; however, in multivariate testing, adjusting for age, sex, and substance use, a history of RH (adjusted odds ratio [aOR]: 3.76, 95% CI 1.27–11.11) was significantly associated with probable PTSD.

**Conclusions:**

RH and PTSD were both exceptionally common in a sample of SSP participants. RH can be detrimental to physical and mental health. Clinicians and policy makers may not consider incarceration as a traumatic experience for PWUD; however, our data suggest that among highly marginalized PWUD, prior exposure to incarceration and RH may add an additional burden to their daily struggles, namely PTSD.

## Introduction

The criminalization of drug use in the United States (US), including severe penalties for use and possession, has had wide-ranging harms for people who use drugs (PWUDs). Criminal-legal involvement can negatively affect housing access, social integration, education, and health status (Cohen et al., 2022). While the US incarceration rate has dropped since its peak in 2010, the US maintains one of the highest rates in the world (358 per 100,000 population in 2020). At any one time over 2 million people are held in US jails or prisons (Carson, 2021; Gramlich, 2021). This mass incarceration has disproportionately affected African American, Latinx, and low-income communities (Harris et al., 2009; Rabuy & Kopf, 2015). There is a well-established link between incarceration and substance use. 65% of the US prison population meets substance use disorder (SUD) criteria. This number grows higher, to 85%, when incorporating people who were under the influence of substances at their time of arrest (National Institute on Drug Abuse, 2020). Evidence-based treatments for SUDs are rarely available in US jails or prisons, and even if substance use decreases during incarceration, return to problematic substance use is common after release from incarceration (Belenko et al., 2013; Winter et al., 2016). Thus, PWUDs are commonly exposed to the criminal-legal system, and this toxic exposure likely impacts the health of PWUD, including mental health.

Post-Traumatic Stress Disorder (PTSD) develops after experiencing, first-hand or vicariously, a traumatic event and involves functionally impairing symptoms of re-experiencing trauma-related memories, avoiding trauma reminders, dysphoric mood, and heightened arousal. The traumatic experiences that precipitate PTSD vary widely and can affect a person at any point in the lifespan (National Institute of Mental Health, 2020). However, intentional interpersonal traumas such as physical assault, rape, and torture have a much greater likelihood of leading to PTSD than accidental or natural events (Breslau et al., 1998; Kessler et al., 1995). PTSD is commonly comorbid with SUDs, with lifetime PTSD prevalence in this population over 50% (Brady et al., 2000; Cottler et al., 1992; Fovet et al., 2021; Najavits et al., 1997). The relationship between PTSD and SUD is likely bidirectional: PWUD can experience violent or traumatic events in conjunction with their substance use, and/or they may use substances, such as alcohol or opioids, to cope with PTSD symptoms (Berenz et al., 2019; María-Ríos & Morrow, 2020). Because PWUD are disproportionately incarcerated, experiences that occur during incarceration may also contribute to the development or exacerbation of both PTSD and SUDs (Kilpatrick et al., 2003).

Aside from exposure to violence, one way incarceration may heighten PTSD risk, including for PWUD, is through exposure to Restrictive Housing (RH) (Andersen et al., 2000; Hagan et al., 2018; Jahn et al., 2022). Commonly referred to as solitary confinement or Special Housing Units (SHU), RH is a method to securely separate individuals in jail or prison from the general population through administrative (non-punitive) or disciplinary (punitive) measures (Federal Bureau of Prisons, 2016; National Institute of Corrections, 2015). Approximately 7.5% of the US federal prison population is housed in RH at any one time, and annually up to 20% of incarcerated individuals experience some type of RH (Beck, 2015). The proportion of current and formerly incarcerated individuals with any lifetime exposure to RH is likely higher. The mechanism by which RH produces negative mental health sequelae is uncertain, but one theory postulates that isolation and lack of stimulation prevents “social reality testing,” which produces severe distress. Human beings require social contact to distinguish whether their perceptions of the environment are real or not (Arrigo & Bullock, 2008). Potentially, these periods of isolation and resulting distress could be followed by posttraumatic stress disorder (PTSD).

Prior studies have suggested an association between prior incarceration, RH, and mental health sequelae, including PTSD symptoms. In a nationally representative sample of Black Americans, those with prior incarceration had significantly more lifetime traumatic episodes and a two times higher rate of PTSD symptoms those without prior incarceration (Anderson et al., 2016). In a US sample of primary care patients who were recently released from prison, exposure to RH during the prior incarceration was strongly associated with PTSD symptoms (Hagan et al., 2018). In a Danish longitudinal cohort study, incident psychiatric disorders were more likely to develop in randomly selected prisoners from RH in comparison to those held in non-RH (Anderson et al., 2000). Other studies have documented significant psychological distress imposed upon persons in RH, and these symptoms could culminate in PTSD during or after incarceration (Grassian, 1983; Haney, 2003). In a US mixed-methods study, people interviewed within two months of entering RH described psychological distress and difficulty accessing health care services (Jahn et al., 2022). A systematic review including 13 studies found moderate evidence that RH was associated with psychological deterioration and two studies documented poor PTSD-related outcomes (Luigi et al., 2020). In contrast, other longitudinal studies have found higher levels of distress among prisoners in RH in comparison to the general population, but they did not find that RH was associated with greater deterioration in well-being during incarceration (O’Keefe et al., 2013; Wright et al., 2023). Thus, some evidence collected during and after incarceration suggests that incarceration and RH may be a traumatic exposure, but there is still uncertainty regarding the impact of RH on mental health.

In this study, we sought to examine the relationship between incarceration history, RH status during incarceration, and PTSD symptoms in a community-dwelling sample of PWUD. We hypothesized that among PWUD, prior exposure to incarceration and RH would be associated with PTSD symptoms and therefore a potentially unrecognized risk factor for PTSD. We recruited participants from syringe services programs (SSPs), community-based programs where PWUD, who are often marginalized from traditional health care venues, can readily engage in diverse health-promoting services (Des Jarlais et al., 2015). While the association between prior incarceration and PTSD has been study in general populations, it has not been well-studied among PWUD.

## Methods

### Setting

Data collection occurred at three SSPs in New York City from June 2021 through March 2022. All three SSPs offered office-based services and street outreach, including case management and referrals to medical care, but they did not have integrated psychiatric services. We chose SSPs as a site for recruitment because they reach a population of PWUD that is different from those recruited from SUD treatment or conventional healthcare settings. Recruitment was carried out by flyers, word-of-mouth, and SSP staff members who referred clients to research staff. Survey responses were collected anonymously. Research staff entered survey responses into an online software (Qualtrics), and individual-level data was kept confidential. Institutional Review Board (IRB) approval and informed consent were received for all parts of the study.

### Participants

A convenience sample included participants who were: (1) 18 + years of age, (2) had current or past opioid use disorder (OUD, self-reported), and (3) received any services at the partner SSP. We targeted participants with OUD specifically, as this study was part of a larger effort to assess the need for and feasibility of combined OUD and PTSD treatment intervention at SSPs.

### Procedure

Trained research staff completed the survey with participants in-person or remotely based on COVID-19 public health restrictions. Data collected included (1) sociodemographic characteristics, (2) substance use over the prior 30 days, (3) self-reported incarceration history, (4) SUD treatment history, and (5) PTSD assessments (i.e., the Life Events Checklist for DSM-5 (LEC-5) and the PTSD Checklist for DSM-5 (PCL-5)). The survey took roughly 30 min to complete. Participants were compensated with $20 cash for completing the survey.

### Measures

#### Trauma exposure and PTSD prevalence/symptom severity

The *Life Events Checklist for DSM-5 (LEC-5)* assesses lifetime traumatic event exposure (Weathers et al., 2013). The LEC-5 consists of 16 potentially traumatic life event categories, such as “Fire or explosion” or “Physical assault (for example, being attacked, hit, slapped, kicked, beaten up)”.

PTSD symptoms were measured with the *PTSD Checklist for DSM-5*, a widely used and well-validated self-report questionnaire (Forkus et al., 2023). The PCL-5 consists of 20 items, aligning with the 20 *DSM-5* symptoms of PTSD. Each item is rated on a five-point scale (0 to 4), with a higher score reflecting more severe symptoms. A PCL-5 cut-point score of 31 or higher was used as a probable PTSD diagnosis, which has been validated as a strong indicator of probable PTSD (Blevins et al., 2015; Bovin et al., 2016).

#### Incarceration

Participants self-reported criminal-legal involvement in several ways. Respondents were asked, “How many months have you been incarcerated in your life?” To determine RH history, we asked participants who were previously incarcerated: “During your time in prison, were you ever placed on restricted status (e.g., solitary, the hold, Seg, AdSeg, the SHU)?”, and if responding yes, reported on, “In your lifetime, what is the total time you spent on restricted status?” Respondents also reported the type of restricted status with responses of: disciplinary action, protective custody, administrative segregation (short-term), chronic discipline, special risk group (SRG), long-term administrative segregation, and special needs. Categories for total time on restricted status in lifetime were: 1 week or less, 1 to 4 weeks, 1 to 3 months, 3 to 6 months, 6 months to 1 year, 1 to 3 years, and 3 years or more. Participants also responded if they were currently on probation or parole.

#### Sociodemographic characteristics

Participants reported age, their racial/ethnic background and if they consider themselves Hispanic or Latinx, their primary language, sex, current gender identity (Male, Female, Transgender, Gender nonconforming), years of education, employment status (employed full-, part-time, or other), and type of health insurance. Current living situation was reported as: own or rent home/apartment, staying at home of family members, staying at home of friend(s)/or someone you know, group home, in a rooming/boarding/halfway house, drug treatment program, shelter, on the street, or other.

#### Substance use

An adapted version of the Addiction Severity Index was used to collect information on substance use during the past 30 days (McLellan et al., 1992). Participants reported the number of days in the past 30 days that they had used cannabis, K2/synthetic cannabis, cocaine/crack, heroin/fentanyl, methadone, buprenorphine, opioid analgesics, benzodiazepines, and amphetamines/stimulants, respectively. For those who responded with 1 or more days of cocaine/crack or heroin/fentanyl use, the route of administration was asked. For those who responded with 1 or more days of methadone, buprenorphine, opioid analgesics, benzodiazepines, and/or amphetamines use, participants responded if the substance(s) was prescribed to them. Participants reported any history of experiencing opioid overdose, defined as being unresponsive or unable to be woken up, waking up in a hospital or ambulance, collapsing or losing consciousness, having difficulty breathing, or having blue skin due to heroin, fentanyl, or another opioid (Siegler et al., 2014). If respondents reported prior opioid overdose, they were also asked how many lifetime overdoses they had experienced.

### Data analysis

We used descriptive statistics to summarize participants’ characteristics. Specifically, we report means and standard deviations for normally distributed continuous variables, medians and interquartile ranges (IQR) for skewed continuous variables, as well as frequencies and percentages for categorical variables. We compared participants with (vs. without) a history of incarceration and those with (vs. without) prior RH status, using T-tests, Mann-Whitney U tests, one-way analysis of variance (ANOVA), or chi square when appropriate, to establish covariates that were associated with the main independent variables. To establish whether prior incarceration and RH status were independently associated with PTSD, we used multivariable logistic regression with a probable PTSD diagnosis as the dependent variable, and a three-level variable reflecting prior criminal legal status (prior incarceration with RH status, prior incarceration without RH status, and no prior incarceration) as the main independent variable. We chose covariates for the model if they had a proposed clinically relevant association with PTSD. These included sex, age, heroin or fentanyl use, other opioid analgesic use, and alcohol use. Interaction analyses were carried out between variables for sex, incarceration history, and having a psychiatric diagnosis other than PTSD. In sensitivity analyses, we used criminal legal status variables that included either the total amount of time (a) incarcerated or (b) held in RH, as well as an alternative PTSD variable with PCL-5 cut-point scores of 33, 34, 38, and 43, which have also been validated as cut-points for probable PTSD diagnosis (Bovin et al., 2016; Dobie et al., 2002; Ibrahim et al., 2018; Murphy et al., 2017).

## Results

153 SSP participants were approached about the study and 139 (91%) were eligible upon screening and completed the study. Participants were mostly male (78%) with a median age of 45 years (Interquartile range [IQR] 38–53), identified as Latinx (55%), were unemployed (81%), and lacked stable housing (65%). Participants reported the following substance use in the past 30 days (median, IQR): heroin or fentanyl (28 days, 3–30), cocaine or crack (15 days, 1–30), alcohol (0 days, 0–5), and benzodiazepines (0 days, 0–5). 58% of participants reported a prior opioid overdose with a mean of 4 overdoses (SD +/- 0.5) among these participants.

Of the 139 total study participants, 109 (78%) had a history of incarceration with 62 (57%) having had a history of RH status while incarcerated. Participants reported a median of 93 months of prior incarceration. The most common type of RH status reported was disciplinary action (92%). Half of those with prior RH reported being in RH for more than 12 months.

Seventy-nine (57%) participants met criteria for probable PTSD (PCL-5 score of 31 or higher). Among all participants, the median PCL-5 score was 31 (IQR 18–51). The most common types of trauma were physical assault (63% of participants), transportation accident (50%), and assault with a weapon (49%). More participants with a history of incarceration had probable PTSD than those without a history of incarceration, but differences were not statistically significant (61% vs. 43%, *p =* 0.092). Those with a history of RH status were more likely to have probable PTSD than those without RH, regardless of incarceration history, but differences were not statistically significant in bivariate testing (65% vs. 51%, *p* = 0.101).

**Table 1:** Participant (*N* = 139) characteristics by incarceration & restrictive housing (RH) history

Characteristic	Sample (*n* = 139)	Never Incarcerated (*n* = 30)	Previously Incarcerated (*n* = 109)	*P* values	Incarcerated with (+) RH (*n* = 62)	Incarcerated with (-) RH (*n* = 47)	*P* values
Age (median, IQR)	45 (38–53)	41 (34–48)	46 (39–54)	0.031	49 (42–55)	43 (35–50)	0.003
Race/ethnicity				NS			NS
Latinx	77 (56%)	17 (57%)	60 (55%)		39 (63%)	21 (45%)	
Non-Hispanic White	28 (20%)	8 (27%)	20 (18%)		8 (13%)	12 (26%)	
Non-Hispanic Black	25 (18%)	4 (13%)	21 (19%)		12 (19%)	9 (19%)	
Male sex	109 (78%)	16 (53%)	93 (85%)	< 0.001	57 (92%)	36 (77%)	0.025
Married	19 (14%)	5 (17%)	14 (13%)	NS	8 (12%)	6 (13%)	NS
Education							
High school graduate/GED or beyond	80 (58%)	22 (73%)	58 (53%)	NS	30 (48%)	28 (60%)	NS
Without employment	113 (81%)	21 (70%)	92 (84%)	NS	54 (87%)	39 (81%)	NS
Unstable housing^a^	91 (65%)	21 (70%)	70 (64%)	NS	45 (73%)	28 (60%)	NS
History of IVDU	97 (70%)	20 (67%)	77 (71%)	NS	44 (71%)	33 (71%)	NS
Median days of substance use past 30 days (IQR)							
Alcohol	0 (0–5)	0 (0–3)	1 (0–5)	NS	1 (0–12)	0.5 (0–4)	NS
Heroin or fentanyl	28 (3–30)	26 (2–30)	28 (3–30)	NS	30 (5–30)	17 (2–30)	NS
Opioid analgesics	0 (0–0)	0 (0–0)	0 (0–0)	NS	0 (0–0)	0 (0–2)	NS
Cocaine or crack	15 (1–30)	15 (0–30)	15 (2–30)	NS	20 (1–30)	14 (3–30)	NS
Benzodiazepine	0 (0–5)	0 (0–3)	0 (0–5)	NS	0 (0–2)	1 (0–13)	NS
History of overdose	81 (58%)	15 (53%)	81 (58%)	NS	39 (63%)	29 (57%)	NS
Median months incarcerated (IQR)	21 (2–120)	0 (0–0)	52 (11–160)	< 0.001	120 (49–211)	12 (3–30)	< 0.001

*Notes* IQR = Interquartile range, IVDU = intravenous drug use, NS = non-significant

^a^unstable housing defined as living: at home of friend/acquaintance, in a group home, in a rooming/boarding/halfway house, at a drug treatment program, in a shelter, or on the street

In multivariable logistic regression, a history of RH status (adjusted odds ratio (aOR) = 3.76, 95% CI 1.27–11.11) was significantly associated with probable PTSD, as was male sex (aOR = 0.36, 95% CI 0.14–0.95). The final model also included age and median days of alcohol, heroin or fentanyl, and other opioid analgesic use in the past 30 days, although these variables were not significantly associated with probable PTSD (Table 2). A history of incarceration without RH (aOR = 2.17, 95% CI 0.79–5.97) was not significantly associated with a positive PTSD screen.

**Table 2:** Multivariable logistic regression model examining probable Post-Traumatic Stress Disorder (PTSD) and incarceration history among syringe services participants (*N* = 139)

Variable	Probable PTSD diagnosis^a^		
	Odds ratio	95% CI	*P* > | z |
Male	0.360	0.137–0.950	0.039
Age (years)	1.004	0.966–1.043	0.841
Alcohol (median days in past 30)	1.000	0.962–1.041	0.981
Opioid analgesics (median days in past 30)	1.053	0.986–1.125	0.121
Heroin or fentanyl (median days in past 30)	1.002	0.973–1.032	0.909
Incarceration with Restrictive Housing^b^	3.761	1.273–11.108	0.017
Incarceration without Restrictive Housing^b^	2.169	0.788–5.970	0.134

^a^ Dependent variable = positive PTSD screen (31 or higher on PTSD Checklist for DSM-5)

^b^ Reference group = no prior incarceration

Interaction testing analyses were carried out between sex, incarceration history, and having a psychiatric comorbidity other than PTSD, none of which were significant when added to models. Sensitivity analyses showed neither length of incarceration or RH status were significantly associated with probable PTSD diagnosis. Additionally, using a PCL-5 cutpoint score of 33 (aOR = 4.10, 95% CI 1.35–12.46), 34 (aOR = 3.95, 95% CI 1.30-12.02), 38 (aOR 3.90, 95% CI 1.28–11.86), and 43 (aOR 4.90, 95% CI 1.49–16.16) to make a probable PTSD diagnosis strengthened the association between RH history and PTSD.

**Fig. 1:**
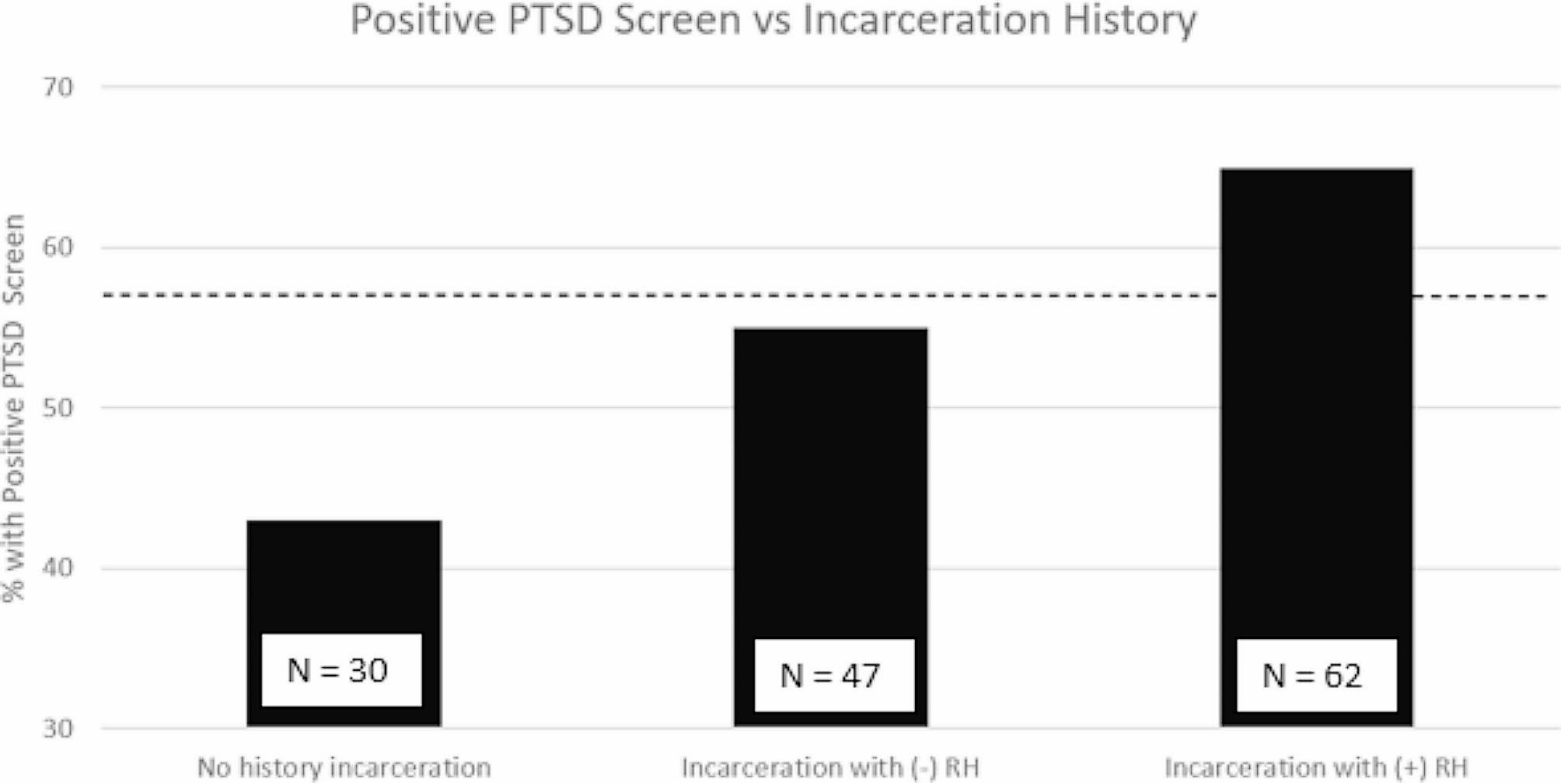
Positive PTSD screen prevalence by incarceration history (*N* = 139). Dashed horizontal line represents the percentage of participants who met criteria for probable PTSD (57%). Number of participants for each group represented by N at base of columns

## Discussion

In this study of PWUD recruited from SSPs, 78% of participants reported prior incarceration and 57% of these participants reported a history of RH. A probable diagnosis of PTSD using a validated screening instrument was common with 57% reporting clinically significant symptoms. Consistent with our hypothesis, there was a significant association between prior RH status and probable PTSD diagnosis in multivariable testing, with previous RH status increasing the odds of a PTSD diagnosis by three-and-a-half times. However, incarceration without being held in RH was not significantly associated with PTSD. Among the many potentially traumatic exposures that PWUD must contend with, exposure to incarceration and RH may be an important one.

Our finding that prior incarceration with RH status is associated with PTSD symptoms among PWUD is consistent with prior studies showing negative mental health consequences from RH. Many studies have documented high levels of psychological distress among people held in RH (Grassian, 1983; Haney, 2003; Reiter et al., 2020; Western et al., 2022). Qualitative interviews with people in RH highlight extreme material deprivation, social isolation, and feeling dehumanized (Western et al., 2022). Other studies have demonstrated an association between RH and self-harming behaviors (Kaba et al., 2014; Reiter et al., 2020). Fewer studies have examined people post-incarceration for long-term sequelae. Hagan et al. (2018) investigated RH and PTSD symptoms in a primary care sample, but all participants were formerly incarcerated, while this study used a comparison group without prior incarceration; however, both studies found that RH was associated with a nearly four times greater odds of screening positive for a clinically meaningful level of PTSD symptoms. Establishing this relationship among PWUD is particularly relevant, because RH can be a punishment for possessing contraband substances or having a positive drug screen during incarceration (Cochran et al., 2018; Lang, 2022). The negative experience of RH could be compounded for PWUD if they experience withdrawal from opioids or other substances while held in RH. Ultimately, though, longitudinal studies that assess PTSD symptoms before and after exposure to RH housing are necessary to extend these findings and draw stronger conclusions regarding causality.

Though the magnitude of the associations is concerning, the cross-sectional methods of this study cannot establish a causal relationship between prior RH and PTSD. Because SUDs are comorbid with PTSD, it is possible that participants’ PTSD symptoms preceded incarceration. A 2022 French study demonstrated that persons entering jail for the first time had higher rates of PTSD than the general population (Fovet et al., 2021). This is similar to US data, which show individuals with PTSD have greater risk for future involvement in the criminal legal system than their non-PTSD counterparts (Donley et al., 2012). During incarceration, behavioral expressions of psychopathology are frequently punished with RH, and persons with mental health conditions are disproportionately placed in RH (Dellazizzo et al., 2020; Reiter et al., 2020; Ryan & DeVylder, 2020). Thus, persons with PTSD are at higher risk for incarceration, and, subsequently, then at a higher risk of being placed in RH. PTSD affects mood, cognition, and arousal states, which may lead to disruptive behavior while incarcerated that are punished with RH. It is also plausible that the directionality of associations leads from RH to PTSD with the conditions of incarceration inflicting psychological harm. While PWUD experience other traumas, such as sexual or physical victimization, our data reflect that incarceration and exposure to RH are also common experiences. People experiencing RH can and do experience significant psychological stressors (Grassian, 1983; Haney, 2003; Reiter et al., 2020; Western et al., 2022). Consequently, RH may cause or exacerbate PTSD. Both potential directions of the association have strong implications for custodial settings.

The first implication is that mental health treatment should be prioritized above punishment for persons with undiagnosed or untreated PTSD who exhibit prohibited behaviors. Screening newly incarcerated individuals for mental health conditions, including PTSD, can create opportunities to initiate treatment or use therapeutic alternatives to RH, which are feasible and potentially impactful. Though the US constitution protects the right to healthcare for people in jail or prison (Alsan et al., 2023), available data suggests that medical and mental health services are limited in most US carceral settings (Curran et al., 2023; Wilper et al., 2009), and RH typically limits access to these services further (Jahn et al., 2022). Nonetheless, the New York City jail system instituted a “Clinical Alternative to Punitive Segregation” initiative for people with serious mental illness who violated jail rules (Glowa-Kollisch et al., 2016). North Carolina’s prison system has also developed a “Therapeutic Diversion Unit” for people with high mental health needs and frequent periods in RH (Remch et al., 2021). Other security-led interventions, such as offering rehabilitative services in RH and providing incentives for compliant behavior, can also reduce the use of RH (Labrecque et al., 2021). A second implication is that if RH causes PTSD in some people, then the practice should be reconsidered all together. The court case, *Madrid v. Gomez*, involving the conditions of RH at Pelican Bay in California, established that solitary confinement of persons with severe persistent mental illness could be considered cruel and unusual punishment and in violation of the Eight Amendment of the US Constitution (Bassett, 2016). Connecticut and New York State recently passed legislation to limit RH use, while in 2024, the US Senate introduced a bill with similar goals (Connecticut Department of Correction, 2021; New York State Senate, 2021; Coons, 2024). The impact of these legislative changes is not yet known.

The high prevalence of PTSD and RH exposure in this urban SSP sample also deserves attention. Participants had spent more than 8 years incarcerated on average, and more than half met criteria for PTSD. PTSD itself is linked with negative health sequelae, ranging from increased incidence of medical comorbidities and drug overdoses to impairments in seeking and accessing SUD treatment (Brady et al., 2000; Goytan et al., 2021; Lee et al., 2020; McCauley et al., 2012). Information on criminal-legal history is sometimes collected in substance use treatment settings (McLellan et al., 1992), but how it is used clinically may vary. Evidence-based PTSD treatment has been adapted for populations with SUDs and criminal-legal involvement (Zielinski et al., 2022). PTSD treatment can be difficult to access for PWUD though. PWUD, especially those utilizing SSPs, face numerous barriers to medical care, including fear of being stigmatized or discriminated against in conventional health care settings. Our participants’ extensive criminal-legal history also may contribute to intersectional stigma faced by PWUD, which can manifest as a barrier to care (Martin et al., 2020). SSP clients often have extended histories of opioid use disorder (OUD), other substance use disorders, high rates of unstable housing, and tenuous support networks when compared to other populations (Craine et al., 2010; Strike & Miskovic, 2018). Our data reflect another layer of marginalization that PWUD and SSP clients must contend with – the long-term consequences of incarceration and RH exposure.

One potential solution to better meet the needs of PWUD with PTSD is to develop integrated care models at trusted community sites such as SSPs. Increasingly, SSPs offer onsite SUD treatment, and integrating evidence-based PTSD care could assure that both issues are addressed (Des Jarlais et al., 2009). Despite their high prevalence and mutually reinforcing harms, care for PTSD and SUDs are rarely considered together, and integrated services for co-occurring disorders are scarcely available in the US (McCauley et al., 2012) There are successful models of integrated SUD and PTSD care, but these would need to be adapted for the SSP setting (Killeen et al., 2011). It is unclear whether prior exposure to incarceration or RH would require unique clinical intervention, but SSPs may be a novel setting to reach a population of PWUD with high needs for clinical services.

Strengths of this study include the unique population, use of a validated screening instrument for PTSD, and detailed data collection on substance use and potential confounding variables. Limitations include convenience sampling, the moderate sample size, recruitment in a single urban area, and reliance on self-reported data (e.g., we were unable to verify the amount of time participants were incarcerated). Additionally, because only a small number of female participants were included in the study, generalizability to the broader population of women who use drugs may be affected. We did not ask participants about levels of social support or PTSD treatment received during or after incarceration. While the estimate of PTSD prevalence at SSPs may differ in other geographic areas, and would be more precise with random sampling, the fact that we were able to easily identify many PWUD with PTSD indicates a substantial unmet treatment need. The cross-sectional study design limits conclusions on directionality of the association, but both possible directions carry important implications. Future research should build upon these preliminary data to establish more precise prevalence estimates, better causal inference, and suggest prevention interventions or integrated treatment approaches.

## Conclusion

Our study highlights a possible, impactful, relationship between current PTSD and prior RH, both of which were common in a sample of SSP clients. RH can be detrimental to physical and mental health. This, often impacts PWUD, who may be placed in RH as punishment for using contraband substances during incarceration, but clinicians and policy makers may not consider incarceration as a traumatic experience for PWUD. As US states experiment with drug decriminalization and alternatives to incarceration for PWUD, good data are needed to compare the relative harms of substance use on individuals and communities with the harms of incarceration and other modes of punishment. Our data demonstrate that among highly marginalized PWUD, exposure to incarceration and RH may add an additional burden to their daily struggles, namely PTSD. Integrated evidence-based SUD and PTSD services are needed for PWUD, but reducing rates of incarceration and use of RH could have a much greater preventative role.

## Data Availability

The datasets used and analyzed during the current study are available from the corresponding author upon reasonable request.
